# Morphological Modification of Metal Oxide Nanomaterials Using Different Types of Organic Modifiers

**DOI:** 10.1002/open.202500342

**Published:** 2025-09-13

**Authors:** Taskiya Akter, Asiful Islam, Abdullah Al Miad, Md. Kawcher Alam, Samina Ahmed, Md. Sahadat Hossain

**Affiliations:** ^1^ Department of Applied Chemistry and Chemical Engineering Noakhali Science and Technology University Noakhali 3814 Bangladesh; ^2^ Institute of Glass & Ceramic Research and Testing Bangladesh Council of Scientific and Industrial Research (BCSIR) Dhaka 1205 Bangladesh

**Keywords:** metal oxides, morphology, nanoparticles, organic modifier

## Abstract

Organic modifiers make incorporating nanoparticles into composite components easier, improving their optical, mechanical, and electrical characteristics. By altering the dimension, arrangement, aggregation, and surface characteristics of the nanoparticles, organic modifiers are thought to be an efficient way to regulate the morphology of nanometal oxides. This can be evaluated by the synthesis and modification of nanometal oxides using various organic agents, such as tiny ligands, various acids, polymers, and so on. Organic modifiers improve crystallinity, bind to oxide surfaces efficiently, and cause morphological changes, reducing agglomeration, raising surface roughness, and exposing more reactive facets according to XRD, FT‐IR, BET, SEM, and TEM investigations. In comparison to unmodified oxides, the results showed that organic modifiers greatly decreased agglomeration, regulated particle size distribution, and improved crystallinity. In general, surface modification with organic modifiers is necessary to maximize the effectiveness of nanoparticles for many reasons, such as materials research, drug delivery, diagnosis, and catalytic processes. In this review, we primarily presented several methods for applying organic modifiers to modify the surface of nanocrystals. Here, we mainly focused on the structural modification of six types of metal oxides (TiO_2_, Fe_3_O_4_, ZnO, Al_2_O_3_, CuO, NiO) via organic modifiers that are commonly used in various nanoparticle‐based applications. The reason behind choosing these six nanoparticles is that they are common in use and there are no such review papers where all the surface modification information is accumulated. The results of this study will assist future researchers in carefully choosing organic modifiers that can be used as a successful method of modifying the morphology of nanometal oxides to satisfy particular functional requirements in technologically sophisticated applications. The importance of organic modifiers in developing nanoparticle technology and propelling advancements in various scientific and industrial fields is also highlighted in this review.

## Graphical abstract

The study reveals the interaction between organic modifiers and metal oxide nanoparticles to tune the structure for improving final applications.

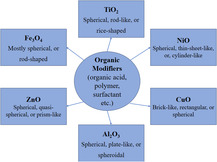

## 1 Introduction

Intentional changing of a crystal's surface characteristics to improve performance, customize it for a particular use, or study its characteristics is known as surface modification of crystal surfaces.^[^ ^1^ ^,^ ^2^ ^]^ Depending on the intended result, several approaches may be used to transform the NP's surface structure's physical, chemical, electrical, or optical properties.^[^ ^3^ ^]^ The nanoparticles of metal oxide (NPs) have been the center of attention in recent years. Nanomaterials have unique properties even when their chemical compositions are the same on a large scale.^[^ ^4^ ^,^ ^5^ ^]^ With a wide range of uses as semiconductors, optical devices, piezoelectric devices, surface acoustic wave devices, sensors, transparent electrodes, solar cells, antibacterial, antioxidant, drug delivery, hypothermia, etc., nano‐metal oxides demonstrate exceptional electrical, magnetic, mechanical, optical, and chemical properties.^[^ ^6^ ^]^ These metal oxide NPs can be composed of a variety of materials, for example, zinc oxide (ZnO), titanium dioxide (TiO_2_), copper oxide (CuO), zirconium dioxide (ZrO_2_), aluminum oxide (Al_2_O_3_), silicon dioxide (SiO_2_), iron oxide (Fe_3_O_4_), magnesium oxide (MgO), nickel oxide (NiO), manganese dioxide (MnO_2_), etc. The distinctive features of metal oxide NPs greatly depend on NP production and dimension.^[^ ^7^, ^8^, ^9^ ^]^ Although certain nanoparticles have good physical and chemical bulk qualities, they do not exhibit adequate surface properties for specific applications. As a result, altering the surface of these materials can be necessary.^[^ ^10^ ^]^ Altering the morphology of these nano‐materials to transfer their enhanced efficiency from the lab to macroscopic practical uses is a significant issue today.^[^ ^11^ ^]^ The primary issue with employing metal oxide nanoparticles is their strong propensity to aggregate, which is brought on because of their increasing ratio of surface area to volume, which raises their surface energy.^[^ ^12^ ^,^ ^13^ ^]^ The NP agglomeration phenomenon causes the particle size to increase, which lowers the surface energy. To solve the issue, metal oxide nanoparticles’ surfaces have been modified using organic modifiers to lower the particles’ surface energy and decrease their propensity to clump together.^[^ ^14^ ^,^ ^15^ ^]^ Metal oxide nanoparticles can effectively and significantly reduce surface energy and aggregation by chemically treating their surfaces.^[^ ^16^ ^]^ It is widely known that the physicochemical properties of nanomaterials (such as TiO_2_, CuO, Fe_3_O_4_, etc.) are reliant on their structure and shape.^[^ ^17^ ^]^ To improve their effectiveness or to tailor their functional, chemical, and controlling crystallite dimensions, size variation, shape, and phase purity are therefore crucial for the production of metal oxide nanoparticles. Common techniques used to modify surfaces include coating, doping, thermal treatment, physical and chemical treatment, and so forth.^[^ ^18^, ^19^, ^20^ ^]^ There are now multiple methods for creating surface‐modified transition metal oxide nanoparticles. Many studies use the breakdown of a metal complex or the dissolution of a metal alkoxide in an organic solvent and surfactant solution.^[^ ^21^ ^,^ ^22^ ^]^ Reverse micelle techniques are also used to create surface‐modified metal oxide nanoparticles, which are then reacted with surfactants in the organic phase. As organic solvents operate as a reaction environment for forming metal oxide and the surface modification of generated nanoparticles, these syntheses are carried out in an organic solvent rather than in an aqueous phase.^[^ ^23^ ^,^ ^24^ ^]^ To improve their effectiveness or to tailor their functional, chemical, and physical properties for specific applications, crystal surfaces must be altered. The mechanical performance, optical action, chemical sensitivity, and ability of the nanoparticles to break down organic contaminants are all enhanced by this method.^[^ ^25^ ^,^ ^26^ ^]^ Modifiers are commonly incorporated into nanoparticles after they are synthesized, changing their qualities by influencing the surface of the nanoparticle by not substantially altering the magnetite's structure.^[^ ^27^ ^]^ Both organic & inorganic modifiers can be used for the structural modification of nanoparticles. Organic modifiers include various polymer compounds, different silane coupling agents, organic acids, surfactants, biomolecules, etc. Organic modifiers, such as amine group‐containing,^[^ ^28^ ^]^ carboxyl group‐containing,^[^ ^29^ ^,^ ^30^ ^]^ and hydroxyl group‐containing,^[^ ^28^ ^,^ ^30^ ^]^ organic chemicals or polymers are commonly employed to produce nanoparticles of metal oxide, which affects their final morphologies and structures. As organic modifiers are compatible with a variety of environmental circumstances, they offer several advantages when utilized in crystal surface modification.^[^ ^31^ ^,^ ^32^ ^]^ After the surface modification of the nano‐metal oxides, the dispersibility is greatly improved, and at the same time, the compatibility between the inorganic nanomaterials and the organic matrix is increased, and the interface problem is reduced.^[^ ^33^ ^]^ It can additionally modify nanoparticles in an organized manner. Nowadays, organic modifiers are the most extensively employed because of their versatility.^[^ ^34^ ^]^ The organic groups’ functionalization is an additional, mainly untapped potential. Functional organic groups on the particle surface may enable intentional contact with molecules, other nanoparticles, surfaces, or substances, whilst basic organic groups are adequate to prevent nanoparticles from clumping together. For example, the interest in metal oxide nanoparticles is also increasing because of their particularly fascinating optical or magnetic properties.^[^ ^35^, ^36^, ^37^, ^38^, ^39^, ^40^, ^41^ ^]^ Depending on the method, substance, and circumstances, the surface modification of nanocrystals can yield a range of structures and morphologies. For example, spherical, rod‐like, rice‐like, hollow structure, brick‐like, flower‐like, and so on.^[^ ^42^, ^43^, ^44^ ^]^ Although several synthetic approaches and surface treatments have been documented in the literature, the majority of these studies either concentrate on particular oxides or single modification routes, which leaves a lack of systematic knowledge regarding the effects of various classes of organic modifiers on morphology and surface characteristics.^[^ ^45^, ^46^, ^47^, ^48^, ^49^, ^50^ ^]^ The study investigates the impact of various organic modifiers, including polymers, acids, surfactants, and small ligands, on the morphology of nano‐metal oxides, aiming to bridge this gap. In contrast to conventional fragmentary claims, this work establishes structure–modifier correlations by comparing their effects on crystal arrangement, dimension of particles, agglomeration, as well as durability. To improve their effectiveness or to tailor their functionality, chemical, and the usefulness of this work lies in providing an overview that explains the mechanics of interactions between modifiers and oxides and shows how these alterations can be intentionally used to enhance operational efficiency.

## 2 Importance of Structural Modification

The structural modification of metal oxides, including TiO_2_, Al_2_O_3_, Fe_2_O_3_, CuO, NiO, and ZnO has significantly enhanced their properties, making them suitable for various applications. There are four primary purposes of surface modification of NPs: 1) to improve or change the dispersion of MNPs; 2) to improve the surface activity of MNPs; 3) to enhance the physicochemical and mechanical properties; and 4) to improve the biocompatibility of MNPs.^[^ ^51^ ^]^ Iron oxide nanoparticles (IONPs) are widely used in nanomedicine; however, they encounter challenges like rapid agglomeration and oxidation, which result in magnetism loss in physiological environments. These limitations arise due to their large surface area, high surface energy, and chemical reactivity. Appropriate surface modification enhances their physicochemical properties, improving biocompatibility and stability.^[^ ^52^ ^]^ Due to their distinctive physical and chemical properties, ZnO NPs are increasingly being explored for biomedical and antibacterial applications. Surface modification is crucial in improving their hydrophobicity and dispersibility in organic media and enhancing compatibility. ZnO has become a key semiconducting metal oxide due to its high optical transparency, tunable electronics, ease of synthesis, low cost, and low toxicity. It also features a large exciton binding energy for optoelectronics, piezoelectric properties for transducers and actuators, high surface sensitivity for sensing, and excellent thermal conductivity and radiation hardness.^[^ ^53^, ^54^, ^55^, ^56^ ^]^ Al_2_O_3_ NPs also have gained significant interest across scientific and industrial fields due to their unique biochemical and physicochemical properties, including high surface area, hardness, thermal stability, biocompatibility, surface functionalization, and electrical insulation. Their nanoscale size allows them to penetrate bacterial and viral cells more effectively, enhancing their antimicrobial efficacy. Al_2_O_3_ NPs are also used in coatings for medical devices and hospital surfaces to prevent bacterial adhesion and biofilm formation, reducing infection risks.^[^ ^57^ ^]^ CuO NPs is a p‐type narrow‐bandgap semiconductor in the transition metal oxide group, have a monoclinic structure, and notable properties such as high thermal conductivity, photovoltaic activity, stability, and antimicrobial effects. Its morphology and particle size significantly impact its electrochemical performance. CuO nanocrystals with cauliflower‐like, nanobelt‐shaped, and feather‐like structures were synthesized via chemical deposition, with different morphologies, which is key in enhancing electrochemical properties. Among them, cauliflower‐like CuO demonstrated the highest specific capacitance (116.9 Fg^−1^), outperforming other morphologies.^[^ ^58^, ^59^, ^60^, ^61^ ^]^ TiO_2_ NPs are known for their bright white color and high refractive index and have been widely used as a white pigment since the 1920s. Its low cost, inertness, and nontoxicity make it ideal for paints, ointments, food coloring, and sunscreens.^[^ ^62^ ^,^ ^63^ ^]^ However, its large band gap and white color limit its effectiveness in photocatalytic applications such as hydrogen generation, CO_2_ reduction, and pollution removal. Extensive research has focused on modifying its electronic and optical properties to enhance photoactivity. Ni and its oxide nanoparticles are used in bright windows to regulate light transmission and solar radiation. NiO NPs also exhibit catalytic properties, making them effective in carbon monoxide oxidation. NiO nanorods demonstrate unique characteristics such as ferromagnetism, optical anisotropy, and applications in particle imaging, photoacoustic imaging, and optical coherence tomography.^[^ ^64^ ^,^ ^65^ ^]^ Using a coupling technique, a novel nano‐SmMn_2_O_5_/Mn_2_O_3_ (SMM) heterostructure coated on porous g‐C_3_N_4_ (CN) nanosheets was created as a possible “nano storage” in the application of electrolytic preservation of hydrogen.^[^ ^66^ ^]^ Silver nanoparticles’ remarkable properties allow for their application in a wide range of industries. Silver salicylate and ammonium metavanadate were used as starting ingredients in the presence of ultrasonic irradiation to create AgVO_3_ micro/nanorods. AgVO_3_ nanorods adorned with AgO nanoparticles were produced in the presence of ethanol, cyclohexanol, DMSO, and acetone, according to SEM and XRD data.^[^ ^67^ ^,^ ^68^ ^]^ Ethylenediamine and citric acid as organic modifiers were used to create graphene quantum dots with a high quantum yield. Long‐term fluorescence stability is demonstrated by these graphene quantum dots.^[^ ^69^ ^]^ A simple combustion process using a variety of carboxylic acids as fuel and capping agents produced the Li_2_CoMn_3_O_8_ nanostructures during the making of a Li‐based battery.^[^ ^70^ ^,^ ^71^ ^]^

## 3 The Synthesis Mechanism of Metal Oxide

From a scientific and technological perspective, metal oxides are the most appealing options among all the functional materials that need to be created at the nanoscale. Metal oxides are the most diverse class of materials due to their special qualities, which encompass nearly every facet of solid‐state physics and materials science.^[^ ^72^ ^]^ For many years, a key component of colloid chemistry has been the synthesis of nanometal oxides with control over size, shape, and size distribution. A mixture of powders reacts directly in the typical and traditional synthesis of oxidic compounds. These solid‐state reactions necessitate high temperatures and small particle sizes in order to bring the reaction partners close enough together and to offer excellent mobility.^[^ ^73^ ^]^ Nevertheless, these severe circumstances hinder the development of thermally labile and metastable solids and prevent fine‐grained control of the processes. Alternative preparation techniques must be created for nanoparticle production, where size and shape play a critical role in defining the attributes.^[^ ^74^ ^]^ The most potential substitutes are soft‐chemistry pathways, which offer minimal processing temperatures, excellent clarity and homogeneity, and strong management from the molecular precursor to the finished product. Not surprisingly, sol–gel chemistry principles, which have a long and fruitful history in the synthesis of bulk metal oxides, have been modified for use in the creation of nanoparticles.^[^ ^75^ ^,^ ^76^ ^]^ Sol‐gel method involves controlled hydrolysis and condensation of precursors. It has mostly been used for TiO_2_, ZnO, and Al_2_O_3_.^[^ ^77^ ^]^ Co‐precipitation is important for magnetic Fe_3_O_4_, requiring careful management of pH and the ratio of Fe^2+^ to Fe^3+^ to prevent oxidation.^[^ ^78^ ^]^ Hydroxide or oxyhydroxide intermediates form under basic conditions and convert to oxides when heated. Hydrothermal or solvothermal synthesis adds pressure‐assisted dissolution and recrystallization mechanisms, resulting in diverse nanostructures such as ZnO rods.^[^ ^79^ ^]^ The solution combustion method is used when speed is necessary. Fuels like urea or glycine are mixed with metal nitrates, which burn in a self‐sustaining reaction to produce oxide nanoparticles within seconds.^[^ ^80^ ^]^ Larger‐scale flame spray pyrolysis quickly produces oxide nanoparticles by spraying precursor solutions into a flame. Vapor‐phase methods, like ALD, enable the growth of thin films for TiO_2_, Al_2_O_3_, and ZnO. They use precursors such as TiCl_4_ or Al(CH_3_)_3_. This approach is particularly useful for coating powders or creating core–shell structures because it deposits oxide layers by layer with angstrom‐level precision by pulsing gaseous precursors in cycles.^[^ ^81^ ^]^

Here is the general mechanism for the metal oxide synthesis:

(1) $$M^{n+}+O\text{source}\overset{\text{Heat}/\text{oxidation}/\text{hydrolysis}}{\to }\text{Mx Oy}+\text{byproducts}$$

## 4 Surface Modification Process by Organic Modifiers

**Figure 1:**
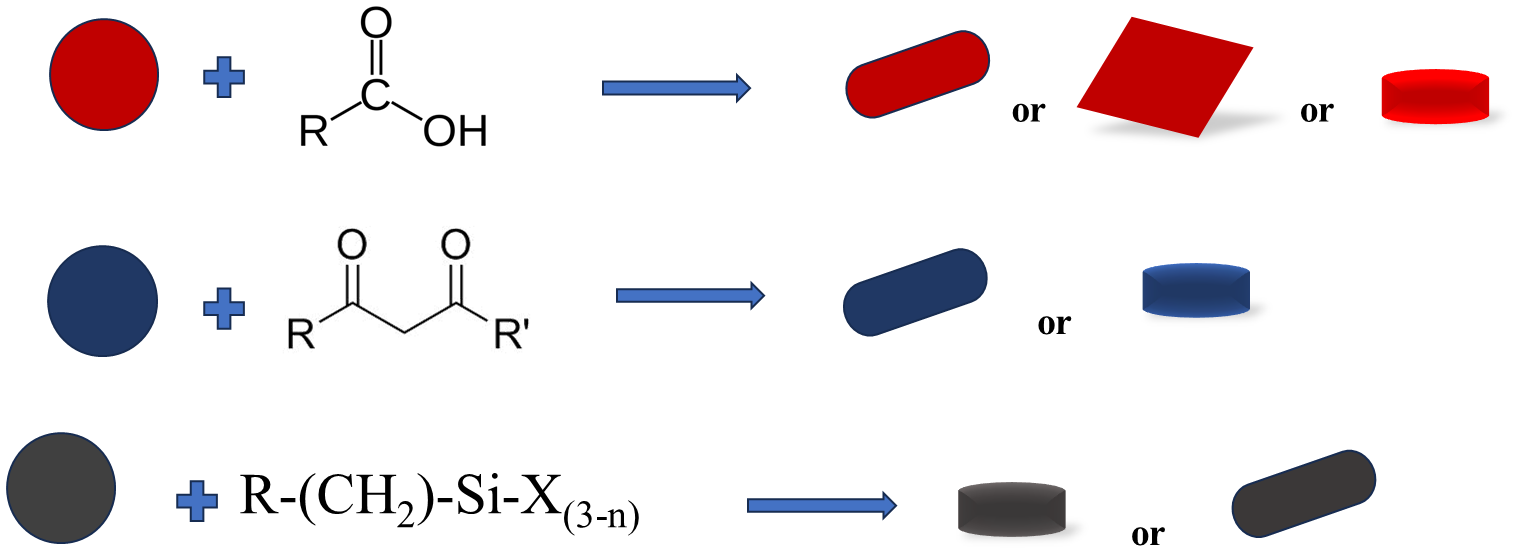
General process for structural modification of metal oxide nanoparticles via organic modifiers.

An organic modifier modifies (a general process is visualized in **Figure** **1**) the crystal surface of nanoparticles by affixing organic molecules to their outermost layer. The surface's solubility, stability, and functionality are all changed by this procedure.^[^ ^82^ ^]^ Functional groups present in the organic modifier can engage with or form bonds with the nanoparticle's atoms on its surface.^[^ ^83^ ^,^ ^84^ ^]^ By forming a barrier around the nanoparticles, the organic molecules can stop them from aggregating and increase their stability in a variety of settings. The chemistry of the exterior can be altered by the connected organic molecules, affecting characteristics like hydrophilicity or hydrophobicity.^[^ ^85^ ^]^

**Figure 2:**
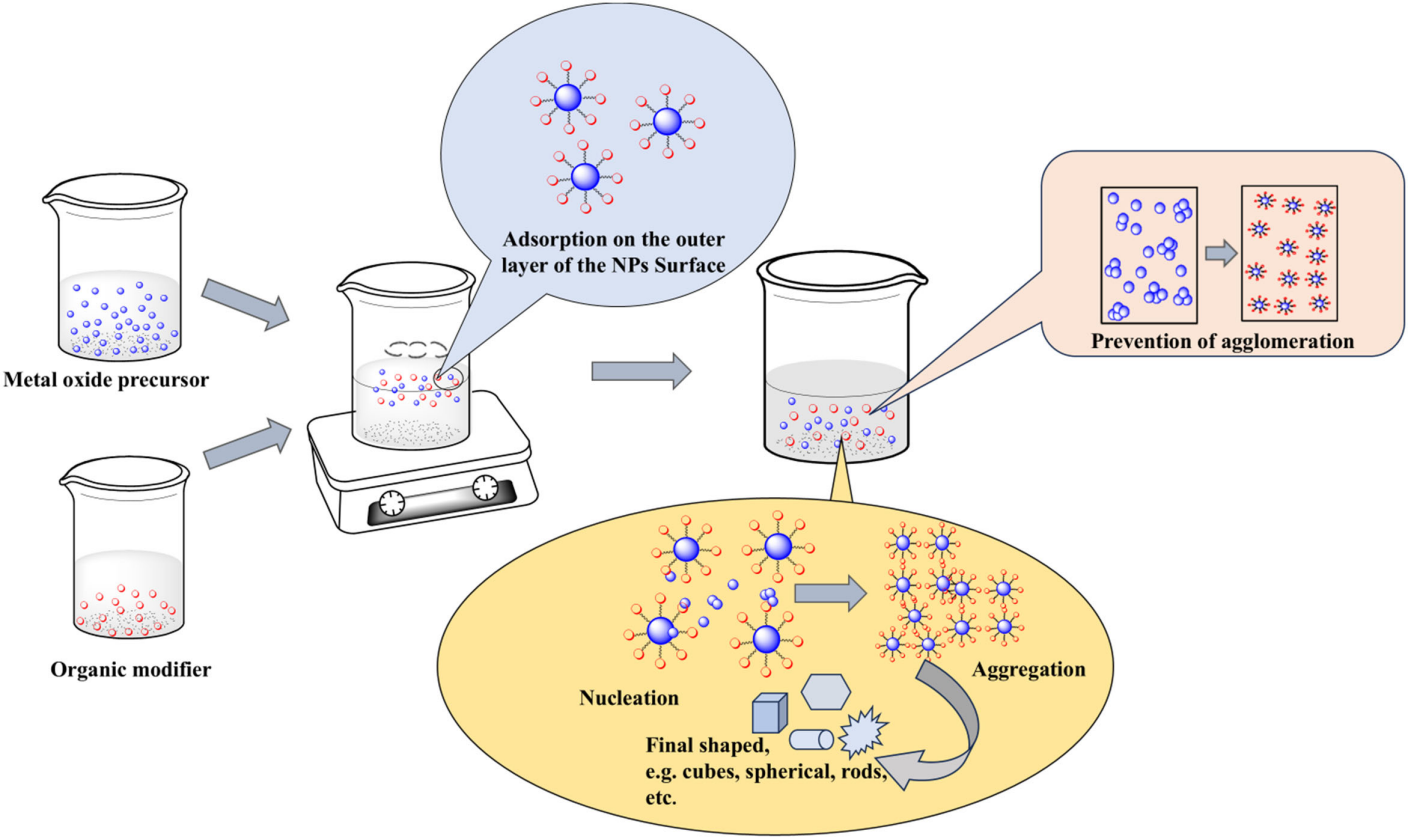
Mechanism for the structural modification of metal oxides. Nanoparticles.

To modify metal oxide nanoparticles, an appropriate modifier must be chosen. Depending on the intended characteristics of the nanoparticles, this modifier may be a tiny organic acid, a polymer, or a surfactant.^[^ ^86^ ^,^ ^87^ ^]^ After that, the appropriate organic modifier is incorporated into a metal oxide precursor mixture. As the modifier reacts with the precursor solution, it influences the metal oxide nanoparticles’ nucleation and growth.^[^ ^88^ ^,^ ^89^ ^]^ The capability of the organic modifier to either limit or promote growth across particular crystallographic orientations determines the ultimate shape and size. The organic modifier directs the development of metal oxide structures with the proper shape and phase by letting the reaction solution grow.^[^ ^11^ ^]^ Organic modifiers control the structure of metal oxide by facilitating the prevention of agglomeration in the medium in which they are dispersed and maintain a spherical shape.^[^ ^90^ ^,^ ^91^ ^]^ The organic modifier compounds are captured on the outermost layer of the metal oxide nanoparticles. Numerous relationships, including hydrogen bonds, covalent bonds, and van der Waals forces, might cause this, depending on the type of modifier and the surface reactivity of the nanoparticles.^[^ ^92^ ^]^ The organic modifier can affect the nanoparticles’ crystal structure.^[^ ^93^ ^]^ For instance, by adhering to particular surfaces preferentially, it might maintain particular crystal facets or stages. By directing the growth of the nanoparticles, the organic modifier can produce a variety of sizes and forms, such as cubes, spheres, or rods.^[^ ^94^ ^,^ ^95^ ^]^ This is accomplished by preferentially adhering to particular crystal faces, preventing or encouraging growth in particular directions (a general mechanism is illustrated in **Figure** **2**). Functional groups present in the organic modifiers alter the colloidal stability of the nanoparticles and dictate their hydrophilicity or hydrophobicity nature.^[^ ^96^ ^]^

## 5 Importance of Six Selected Nanoparticles (TiO_2_, CuO, NiO, Fe_3_O_4_, ZnO, and Al_2_O_3_)

Metal oxide nanoparticles (TiO_2_, CuO, NiO, Fe_3_O_4_, ZnO, and Al_2_O_3_) are extensively researched due to their multifunctionality in electronics, medicine, energy, catalysis, environment, and industry. Their nanoscale size enhances their surface area, reactivity, and performance, making them superior to their bulk counterparts in various applications. Copper is an essential micronutrient crucial for plant health, incorporated into various proteins and enzymes. Copper nanoparticles have garnered considerable interest in future nanodevices owing to their superior electrical conductivity, catalytic properties, and surface‐enhanced Raman scattering activity. These materials find extensive applications in antimicrobials, gas sensors, electronics, textile coatings, batteries, solar energy conversion, and high‐temperature superconductors.^[^ ^97^ ^,^ ^98^ ^]^ Alumina nanoparticles hold great potential for detecting infections, delivering antimicrobial drugs, and combating antibiotic‐resistant pathogens.^[^ ^99^ ^]^ The ZnO semiconductor demonstrates distinctive characteristics, such as excellent transparency, significant electron mobility, a broadband gap, and robust luminescence at room temperature. Its nontoxic nature makes it an effective photocatalyst for the degradation of environmental pollutants. ZnO bulk and thin films exhibit significant sensitivity to a range of toxic gases.^[^ ^100^ ^,^ ^101^ ^]^ Magnetite (Fe_3_O_4_) nanoparticles (NPs) are compelling nanomaterials in material science, chemistry, and physics due to their advantageous features, including soft ferromagnetism, half‐metallicity, and biocompatibility. The three most commonly investigated biomedical uses of Fe_3_O_4_ nanoparticles are magnetic contrast agents, hyperthermia, and drug delivery.^[^ ^102^ ^]^ TiO_2_ nanoparticles are utilized as photosensitizing agents for cancer treatment and photodynamic inactivation of antibiotic‐resistant microorganisms. They, together with their composites and hybrids, find usage as excipients in pharmaceuticals, in sunscreens for cosmetics, as colorants in white plastics, and as a cost‐effective, nontoxic food coloring.^[^ ^103^ ^]^ NiO nanoparticles are affordable, nontoxic, and highly stable conductive materials. They are employed in medical applications like imaging, medication delivery, biological detection, and antibiotics. Additionally, NiO NPs effectively remove organic and inorganic contaminants, contributing to environmental protection.^[^ ^104^ ^]^

Here are some generalized structural modification reactions for the discussed metal oxide nanoparticles in this review.

(2) $${\mathrm{TiO}}_{2}\;\left(\mathrm{unmodified}\right)+R-\mathrm{Si}{\left(\mathrm{OR}\prime \right)}_{3}\to {\mathrm{Modified}\;\;\mathrm{TiO}}_{2}\;+3R-\mathrm{OH}$$

(3) $${\mathrm{Fe}}_{3}\;O_{4}\;\left(\mathrm{unmodified}\right)+R-\mathrm{COOH}\to {\mathrm{Modified}\;\;\mathrm{Fe}}_{3}\;O_{4}\;+H_{2\;}O$$

(4) $$\mathrm{Unmodified}\;\;\mathrm{ZnO}+R-{\mathrm{PO}}_{3\;}H_{2}\to \mathrm{Modified}\;\;\mathrm{ZnO}+H_{2}\;O$$

(5) $${\mathrm{Unmodified}\;\;\mathrm{Al}}_{2\;}O_{3}\;+R-\mathrm{NCO}(\mathrm{organic}\;\;\mathrm{modifier})\to \mathrm{Modified}\;{\mathrm{Al}}_{2}O_{3}$$

(6) $$\mathrm{CuO}\left(\mathrm{unmodified}\right)+C_{18}\;H_{34\;}O_{2}\;\to \mathrm{CuO}\left(\mathrm{modified}\right)+H_{2}\;O$$

(7) $$\mathrm{NiO}\left(\mathrm{Unmodified}\right)+C_{12}\;H_{25\;}{\mathrm{OSO}}_{3}\;\mathrm{Na}\to \mathrm{NiO}\left(\mathrm{Modified}\right)+{\mathrm{Na}}^{+}$$

## 6 Titanium Dioxide (TiO_2_)

Titanium dioxide, often known as TiO_2_, titania, or titanium (IV) oxide, is an inorganic material with a wide range of real‐world uses.^[^ ^105^ ^,^ ^106^ ^]^ The fine white powder, frequently called "titanium white," is mostly employed as a pigment due to its hiding power (opacifying strength) and brightness. Due to its chemical resistance, thermal stability, and UV protection abilities, TiO_2_ is a vital pigment in paints, coatings, plastics, and paper.^[^ ^107^ ^]^ The global production of titanium dioxide is experiencing a steady increase.^[^ ^108^ ^]^ There are three main crystal structures or forms that titanium dioxide (TiO_2_) can take: anatase, rutile, and brookite. Comparing three crystal structures, we found that Rutile exhibits superior thermal stability compared to brookite and anatase, transforming rutile when subjected to temperatures exceeding 800 °C.^[^ ^109^ ^]^ This phase transition highlights the rutile's resilience under high thermal conditions, as it is a thermodynamically favored polymorph in such environments.^[^ ^110^ ^]^ TiO_2_ NPs can be made chemically and physiologically from green plants, bacteria, and fungal extraction. Various methodologies exist in the manufacturing of TiO_2_ nanoparticles, involving the sol–gel technique,^[^ ^111^ ^]^ solvothermal technique,^[^ ^112^ ^]^ hydrothermal technique,^[^ ^113^ ^]^ electrochemical process,^[^ ^114^ ^]^ co‐precipitation strategy,^[^ ^115^ ^]^ etc. However, these methods possess drawbacks; like, the sol–gel process needs hours to days for the synthesis of TiO_2_ nanoparticles, the process of hydrothermal process is synthetic and entails chemical reactions, and the precipitation method presents challenges in controlling particle size, as rapid precipitation results in the creation of bigger particles. The synthesis of microbial nanoparticles is environmentally sustainable and cost‐effective, as it operates under mild conditions.^[^ ^116^ ^]^ The distinct characteristics of TiO_2_ nanostructures, resulting from their particular surface area and quantum size effects, make them very suitable for many applications that include photochemical catalysis, photoelectrochemical systems, dye‐sensitized solar cells, and gas sensors.^[^ ^117^, ^118^, ^119^, ^120^, ^121^ ^]^ Abbas et al. did a study in which TiO_2_ produced by the sol–gel method, highly reactive metal alkoxides, is used as a starting material. Adding modifiers such as acetylacetone, acetic acid, or other complex chemical ligands can control the reactivity of metal alkoxides, which are needed to obtain sols and gels with the required properties. They form new molecular precursors by molecular‐level chemical reactions with alkoxides. In sol–gel manufacturing, these altered alkoxide precursors can improve control of the hydrolysis‐condensation process.^[^ ^122^, ^123^, ^124^ ^]^ Zhou et al. conducted a modification process for TiO_2_ by two types of organic modifiers, e.g., sodium stearate and sodium oleate, where the calcination temperature was maintained at 65 °C. Modified nanoparticles were found to be hydrophobic. The modification reaction takes place as follows.^[^ ^125^ ^]^

(8) $${\mathrm{TiO}}_{2}\left(\mathrm{unmodified}\right)+C_{17}H_{35}\mathrm{COONa}\left(\mathrm{sodium}\;\mathrm{stearate}\right)\to {\mathrm{TiO}}_{2}\left(\mathrm{modified}\right)\cdot C_{17}H_{35}{\mathrm{COO}}^{-}+{\mathrm{Na}}^{+}$$

(9) $${\mathrm{TiO}}_{2}\left(\mathrm{unmodified}\right)+C_{17}H_{33}\mathrm{COONa}\left(\mathrm{sodium}\;\mathrm{oleate}\right)\to {\mathrm{TiO}}_{2}\left(\mathrm{modified}\right)\cdot C_{17}H_{33}{\mathrm{COO}}^{-}+{\mathrm{Na}}^{+}$$

Another study uses acetic acid as a modifier due to its ready dissolution of a diverse range of precursor molecules, facilitating the formation of several multi‐cation solutions.^[^ ^126^ ^]^ Precipitation happens quickly when water is introduced to Ti(OPri)_4_. However, when acetic acid is present, uniform and limpid TiO_2_ gels are formed. When acetic acid is introduced to Ti alkoxides, a highly exothermic reaction occurs, creating a novel molecular precursor.^[^ ^127^ ^,^ ^128^ ^]^ Choi et al. adopted an encapsulation approach to modify the surface of TiO_2_ NPs with the help of polystyrene, aiming to enhance the uniform dispersion of the NPs within the PVC matrix while minimizing agglomeration.^[^ ^129^ ^]^ Nano‐additive treatment led by the polymer utilized for the surface treatment should be thermodynamically miscible and compatible with the host polymer matrix.^[^ ^130^ ^]^ TiO_2_ surfaces that have previously had Al_2_O_3_ treatment have been successfully surface‐treated using polyhedral oligomeric silsesquioxanes (POSS) molecules. By reducing nanoparticle aggregation, POSS increased TiO_2_ dispersion and allowed for more UV absorption.^[^ ^131^ ^]^ Polyaniline (PANI) is a promising polymer for surface modification of TiO_2_ nanoparticles due to its simple production, inexpensive monomer, environmental stability, and inherent redox interactions.^[^ ^132^, ^133^, ^134^ ^]^ Ali et al. revealed that the morphology of TiO_2_ NPs is a surface modification by PANI and was examined using TEM and SEM analysis. Micrographs obtained from TEM and SEM were analyzed for both unmodified TiO_2_ NPs and PANI‐TiO_2_ NPs to evaluate the impact of surface modification on their morphology and degree of aggregation.^[^ ^135^ ^,^ ^136^ ^]^ SEM analysis reveals that unmodified TiO_2_ NPs exhibit significant aggregation due to their high surface energy, whereas the aggregation of PANI‐TiO_2_ NPs is drastically reduced.^[^ ^137^ ^]^ The reduction in the agglomeration of the PANI‐TiO_2_ nano‐additive is evident from its smoother surface topographical curves, in contrast to the irregular surface topographical curves seen in the unmodified TiO_2_ nano‐additive.^[^ ^138^ ^]^ The findings suggest that coating TiO_2_ NPs with PANI chains is an effective method for reducing nanoparticle agglomeration.^[^ ^136^ ^]^ In the last two decades, studies have focused on the surface treatment of industrial TiO2 (Degussa P25, *d* = 30 nm) using benzene derivatives, specifically catechol, and salicylic acid.^[^ ^139^, ^140^, ^141^, ^142^ ^]^ Jankovic et al. modified TiO_2_ nanoparticles with a vast class of enediol ligands and compared them with unmodified TiO_2_. Enediol ligands like 2,5‐dihydroxybenzoic, salicylic, 3,4‐dihydroxybenzoic acid, 2,3‐dihydroxybenzoic, and catechol can alter the coordination structure of the outermost Ti atoms, resulting in a displacement of the absorption onset into the visible spectrum. The attachment of the modifier molecules to undercoordinated surface Ti atoms (defect sites) causes a considerable shift in the effective band gap and the start of absorption. By selecting the appropriate ligand, TiO_2_'s electrical properties can be fine‐tuned.^[^ ^143^ ^]^ It is commonly recognized that a nanomaterial's form and structure affect its physicochemical properties.^[^ ^17^ ^]^ Controlling crystallite size, size distribution, structure, and phase purity is, therefore, essential for the synthesis of nanosized TiO_2_. To maintain the final morphologies and therefore the characteristics of anatase TiO_2_ nanoparticles, organic modifiers, such as amine group‐containing,^[^ ^144^ ^]^ carboxyl group,^[^ ^145^ ^]^ and hydroxyl group‐containing^[^ ^144^ ^,^ ^145^ ^]^ organic compounds or polymers are frequently utilized in the manufacturing process. Investigating how organic modifiers affect the morphology of rutile TiO_2_ nanoparticles produced hydrothermally has, however, received significantly less attention. In order to create the desired product with the required geometries and interface structures, these methods use organic components with different functional groups to cooperate with the material's crystal structure.^[^ ^146^ ^]^ According to a study by Peng et al., when citric acid was utilized as a modifier instead of glycerol, the particle sizes for nanosized TiO_2_ were narrower and smaller.^[^ ^147^ ^]^ Citric acid, which contains carboxylic groups, likely creates stronger interactions with the TiO_2_ surface, leading to narrower distributions and smaller nanoparticles compared with glycerol.^[^ ^148^ ^,^ ^149^ ^]^ The resulting TiO_2_ nanoparticles had smaller average particle sizes and narrower particle size dispersion when two carboxylic acid groups were utilized as modifiers rather than three.^[^ ^145^ ^,^ ^150^ ^]^ For nanosized TiO_2_ synthesis, using formic, acetic, and dodecanoic acid (increasing carbon chain length) decreased the average particle size and distribution. A study involved coating TiO_2_ through dopamine polymerization to enhance its photocatalytic performance.^[^ ^151^ ^]^ To maintain the pH of the solution above, 0.20 g of HMTA was added after 0.16 g of TiO_2_ had been distributed in 38 mL of water, followed by the addition of 0.0153 g of DA. After stirring the mixture for 2 h at an ambient temperature, the solution gradually transitioned to a pale‐yellow color. It was subsequently centrifuged at 10,000 rpm for 2 min and thoroughly rinsed with deionized water and ethanol. After natural drying, a solid powder (dark yellow colored) was obtained, referred to as TiO_2_@PDA‐0.4 mg mL^−2^. By maintaining the quantities of HMTA and TiO_2_ constant, different TiO_2_@PDA products can be synthesized by varying the DA concentration and stirring duration.^[^ ^152^ ^,^ ^153^ ^]^ The TEM analysis of TiO_2_ NPs, both pre‐and post‐PDA coating, exhibited little aggregation of TiO_2_, distinguished by sharp edges and well‐defined borders. Due to PDA's excellent adhesion quality, the aggregation of TiO_2_@PDA‐0.4 mg mL^−2 ^ h^−^^1^ strengthened slightly after the PDA surface coating. Consequently, the sharp corners and edges of PDA‐coated TiO_2_ will be obscured.^[^ ^152^ ^]^ In a study, the contact angle of TiO_2_ gradually increases with higher doses of modifiers when modified with sodium oleate and sodium stearate.^[^ ^154^ ^]^ The contact angles reach 125.6° for TiO_2_ modified with sodium stearate and 121.3° for TiO_2_ modified with sodium oleate. Since these values exceed 90°, they mark a critical shift from hydrophilicity to hydrophobicity at a modifier dosage of 1.5%. The unmodified TiO_2_ initially has a contact angle of 7°, indicating a strongly hydrophilic surface. After modification, the TiO_2_ surface transitions to a hydrophobic state.^[^ ^16^ ^,^ ^155^ ^]^ However, as the dosage is increased further, the wetting contact angle on the TiO_2_ surface decreases into a minimal range. The findings show that both modifiers can alter the TiO_2_ surface's wettability.^[^ ^125^ ^,^ ^156^ ^]^ Few recent works on the structural modification of TiO_2_ are registered in **Table** **1**.

**Table 1:** Surface modification data of TiO_2_ nanoparticles via different organic modifiers.

Reagent	Modifier	pH	Morphological Structure	XRD	SEM	TEM	Calcination Temp. [°C]	References
				[nm]				
TiO_2_ NPs, methanol	Bioactive diacid	–	Spherical	21–24	–	30 or less	–	^[^ ^272^ ^]^
Titanium (IV) tetra‐isopropoxide and All‐trans‐retinoic acid, titanium (IV) nbutoxide (99%)	Carboxylic acid	>3.9	Amorphous	≈0.7	–	–	–	^[^ ^273^ ^]^
Ti{OCH(CH_3_)_2_}_4,_ C_2_H_5_OH	Acetylacetone	–	Crystalline spherical	20–30	20–25	–	400	^[^ ^274^ ^]^
Ti{OCH(CH_3_)_2_}_4_	Acetic acid	–	Crystalline spherical	20–30	25–30	–	400	^[^ ^274^ ^]^
H_2_TiO_3_, H_2_SO_4_(98%), HCl(38%), CH_3_CH_2_OH, NaCO_3_, activated carbon	Citric acid	0.4	Crystalline rodlike	–	–	L: 69.2D: 31.4	220	^[^ ^275^ ^]^
H_2_TiO_3_, H_2_SO_4_(98%), HCl(38%), CH_3_CH_2_OH, NaCO_3_, activated carbon	HCOOH	0.4	Crystalline rodlike	–	–	L: 56D: 27.1	220	^[^ ^275^ ^]^
[Ti(OCH(CH_3_)_2_)_4_	Acetic acid	3.4–3.5	Crystalline rod shape and rice shape	6	–	≈ 5	–	^[^ ^269^ ^]^
Ti(OCH(CH_3_)_2_), C_6_H_5_CH_3_	C_10_H_16_N_2_O_8_	8	Crystalline spherical	–	31.7, 46.3	W: 6L: 50	450	^[^ ^276^ ^]^
TiO_2_ powder, CH_3_OH	–	–	Crystalline	21–24	–	30	400	^[^ ^272^ ^]^
Epoxy resin, MMA, styrene, sodium ethyl xanthate, and 3‐(chloropropyl) triethoxy silane	Di‐block copolymer	–	Amorphous	–	–	–	80	^[^ ^277^ ^]^
TiO_2_ nanotube, HCl, NaOH	3‐aminopropyl triethoxysilane (APTS)	7	Crystalline rod like	–	–	W: 9.83–13.4L: 138–140	400	^[^ ^278^ ^]^
TiO_2_ NPs, ethyl acetate, chloroform	Hexanoic acid	–	Crystalline narrow spherical	–	–	3.7	25	^[^ ^279^ ^]^
TiO_2_ NPs, ethyl acetate, chloroform	n‐hexylamine	–	Crystalline narrow spherical	–	–	3.5	25	^[^ ^279^ ^]^
TiO_2_/Ag+ NPs, CH_3_SO_3_H, toluene, triethylamine	APS	–	Spherical	–	–	70	80	^[^ ^280^ ^]^

## 7 Iron Oxide (Fe_3_O_4_)

From a research and development standpoint, Nanometer‐sized magnetic particles represent a novel material of tremendous interest.^[^ ^157^ ^]^ This category of intriguing nanomaterials, commonly known as magnetic nanoparticles (MNPs), has been thoroughly researched for several scientific uses.^[^ ^158^, ^159^, ^160^ ^]^ MNPs are used in various fields such as magnetic separation, magnetic labeling, sensing technologies, memory storage devices, and catalytic processes.^[^ ^161^, ^162^, ^163^ ^]^ Applications for magnetic nanoparticles (MNPs) in biomedicine include generating heat to treat hyperthermia, creating contrast responses for magnetic imaging, and enabling remote control of targeted medication administration.^[^ ^130^ ^,^ ^131^ ^]^ One particular kind of magnetic nanoparticle that has been thoroughly studied for its many industrial and scientific applications, such as MRI and data storage, is iron oxide nanoparticles (IONPs). Wustite (FeO), mixed iron (II, III) oxide (Fe_3_O_4_, magnetite), and iron (III) oxides are among the various types of iron oxides. These include epsilon phase (e‐Fe_2_O_3_), alpha phase (a‐Fe_2_O_3_, hematite), gamma phase (γ‐Fe_2_O_3_, maghemite), and beta phase (b‐Fe_2_O_3_).^[^ ^164^ ^]^ As they have characteristics like inexpensive cost, low toxicity, and excellent magnetic characteristics, iron oxide nanoparticles (Fe_3_O_4_ NPs) have become the most well‐known and widely used form of magnetite nanomaterials.^[^ ^165^ ^]^ In most cases, the size and form of magnetic nanoparticles dictate their chemical and physical characteristics.^[^ ^166^ ^]^ There are currently many established methods for producing magnetic Fe_3_O_4_ nanoparticles, including polyols,^[^ ^167^ ^]^ microemulsions,^[^ ^168^ ^]^ laser pyrolysis,^[^ ^169^ ^]^ sonochemical synthesis,^[^ ^170^ ^]^ and chemical co‐precipitation,^[^ ^171^ ^]^ among others. Fe^2+^ and Fe^3+^ ion activity with OH^−^ is necessary for the formation of Fe_3_O_4_ nanoparticles. Modifiers are frequently employed to manufacture nanoparticles of Fe_3_O_4_ to inhibit agglomeration and alter their characteristics. Organic acids and salts, including tartaric acid, citric acid, sodium citrate, oleic acid, and sodium oleate, are among the often‐encountered modifiers. Furthermore, other organic substances, including polyvinyl alcohol (PVA) and dextran, as well as water‐soluble polymers like polyethylene glycol (PEG), PEG diacid, and polyvinylpyrrolidone (PVP), are frequently utilized. After being synthesized, modifiers are commonly applied to nanoparticles, changing their characteristics by influencing the surface of the nanoparticle without substantially altering the magnetite's structure.^[^ ^172^, ^173^, ^174^, ^175^ ^]^ In one study, sodium citrate (Na_3_C_6_H_5_O_7_), polyvinylpyrrolidone (PVP), dextrin (C_6_H_10_O_5_), and ethylene glycol (C_2_H_6_O_2_) were employed directly as reaction modifiers to create Fe_3_O_4_ nanoparticles using the co‐precipitating approach. Modifiers can produce nanoparticles that are smaller than those without the Fe_3_O_4_ modification. The solution produced the tiniest nanoparticles when tartaric acid was added as a modifier. The magnetite generated in the solution containing PVC exhibited the least amount of nanoparticle size change, and the shape of the synthesized nanoparticles of magnetite nanoparticles was described using TEM. Pure magnetite nanoparticles (Fe_3_O_4_) without a modifier had a spherical shape, whereas nanoparticles synthesized with PVP and ethylene glycol as modifiers were reported to have a similar morphology. The Fe_3_O_4_‐tartrate and Fe_3_O_4_‐citrate components produce a strongly agglomerated structure. By using dextrin as a modifier, a composite with spherical magnetite nanoparticles dispersed throughout a polysaccharide matrix could be created.^[^ ^27^ ^]^ Mustafa et al. conducted a study on iron oxide NPs fabricated using a one‐pot electrodeposition procedure, employing ethylenediaminetetraacetic acid (EDTA) as a capping agent. EDTA was selected for its ability to suppress crystal grain formation during the production of Fe_3_O_4_ particles with various diameters. Moreover, EDTA's carboxylate (COO–) groups have a high coordination affinity with the Fe^3+^/Fe^2+^ cations on the Fe_3_O_4_ surface. Both uncoated and EDTA‐functionalized magnetic nanoparticles (MNPs) have a pure Fe_3_O_4_ crystalline structure, according to XRD analysis. According to morphological analyses carried out with the TEM and FE‐SEM, the EDTA‐coated MNPs are made up of spherical particles with an average size of 15 nm. TEM also revealed that EDTA‐coated NPs exhibit improved dispersion and less aggregation. Since the EDTA capping layer on the Fe_3_O_4_ surface inhibits particle agglomeration during the deposition process, EDTA's function as a coating agent during the creation and growth of magnetite particles is responsible for this enhanced dispersion.^[^ ^176^ ^]^ The surface chelation process of Fe_3_O_4_ via EDTA takes place as follows:

(10) $${\mathrm{Fe}}^{3+}+{\mathrm{EDTA}}^{4-}\to \mathrm{Fe}{\left(\mathrm{EDTA}\right)}^{-}$$

(11) $${\mathrm{Unmodified}\;\mathrm{Fe}}_{3}O_{4}+-{\left[{\mathrm{CH}}_{2}-\mathrm{CH}\left(\mathrm{COOH}\right)\right]}_{n^{-}}\to {\mathrm{Modified}\;\mathrm{Fe}}_{3}O_{4}\;\mathrm{structure}$$

Shin et al. did a study where polyacrylic acid‐modified Fe_3_O_4_ nanoparticles (300 nm) were synthesized via the polyol method. PAA solutions (100 mL) with a concentration of 1,3,5,10 wt% were created for Fe_3_O_4_ surface modification by dissolving in distilled water and stirring at 300–600 rpm. After surface modification, the average sizes were 256 nm for 10 wt%, 202 nm for 5 wt%, and 340 nm for 1 wt%. As the PAA content increased, the particle size distribution narrowed, and the peak shifted toward smaller sizes. The PAA modification improved particle dispersion by reducing the zeta potential through surface charge effects. However, at 10 wt% PAA, excess polymer caused particle agglomeration due to clumping.^[^ ^177^ ^]^ For diagnostic applications, a study produced uniform magnetic nanoparticles (Fe_3_O_4_‐DPA‐PEG‐COOH) coated with a water‐soluble polymer. The co‐precipitation method was employed to create superparamagnetic Fe_3_O_4_ nanoparticles. Fe_3_O_4_ nanoparticle surface modification using a polymer chain proved to be more fruitful. The altered nanoparticles were utterly steady, easily dispersed in water, and superparamagnetic. According to an analysis of the outcomes of several tests, including MNP concentration, echo time TE value, cytotoxicity effect, and cellular uptake on MDA‐MB‐231 breast cancer cells, PEG diacid‐grafted Fe_3_O_4_ nanoparticles were the best contrast agent for magnetic resonance imaging (MRI). The nanoparticles’ negative surface charge (−27.69 to −35.13 mV) could be due to the presence of the polymer's end carboxyl groups on their surface.^[^ ^145^ ^]^ Alginates are anionic polysaccharides that contain β (1–4) linked D‐mannuronic acid and α (1–4) linked L‐guluronic acid components.^[^ ^146^, ^147^, ^148^, ^149^ ^]^ Three steps are typically involved in the manufacture of alginate‐coated nanoparticles of iron oxide. Alginate is gelled in a ferrous ion solution in the first phase, ferrous ions are precipitated in situ by alkaline treatment in the second phase, and ferrous hydroxide is oxidized using oxidizing agents such as O_2_ or H_2_O_2_ in the third phase.^[^ ^150^ ^]^ Alginate‐coated magnetic iron oxide nanoparticles have also been produced using a two‐step coprecipitation process. By coprecipitating ferric and ferrous ions using the alkaline treatment and alginate coating, this technique produces Fe_3_O_4_ particles.^[^ ^81^ ^]^ The core diameter of the MNPs produced using this method was found to be between 5 and 10 nm. The hydrodynamic diameter of the MNPs after alginate coating was determined to be between 193.8 and 483.2 nm.^[^ ^151^ ^]^ A study is being conducted to examine citrate‐capped nanoparticles of superparamagnetic iron oxide for the traditional cancer therapy technique of heating tumors (hyperthermia). Fe^2+^ and Fe^3+^ ions were co‐precipitated with NH_4_OH to create magnetite nanoparticles. XRD confirms the presence of crystalline cubic spinel superparamagnetic iron oxide nanoparticles. At roughly 10 nm, the TEM images show spherical particles with a small range of sizes. The MNP‐CAs showed superparamagnetic behavior ideal for hyperthermia treatment. This indicates that they are very magnetically sensitive (74 emu g^−^^1^), which is necessary for producing heat in a magnetic field. Furthermore, their aptitude for hyperthermia is further supported by their homogeneous particle size and absence of magnetic remanence, or magnetic memory.^[^ ^178^ ^]^ Utrica is a unique green precursor that can be used to prepare magnetic NiFe_2_O_4_ rod‐like platelets. After being exposed to a frequency magnetic field for 48 h, the prepared nanoparticles showed increased cytotoxicity effects.^[^ ^179^ ^]^ Few recent works on the structural modification of Fe_2_O_3_ are listed in **Table** **2**.

**Table 2:** Surface modification data of Fe_3_O_4_ nanoparticles via different organic modifiers.

Reagent	Modifier	pH	Morphological structure	XRD	SEM	TEM	Calcination Temp. [°C]	References
				[nm]				
FeCl_3_^.^ ·6H_2_O, FeCl_2_^.^ ·4H_2_O, 25% aq. Ammonia, 37% HCl	Sodium citrate, EDTA	–	Spherical	14	–	12	90	^[^ ^281^ ^]^
FeCl_3_, NaOH, FeSO_4_^.^·7H_2_O	Polyvinylpyrrolidone	–	Crystalline spherical	D: 9.2	–	.252	50	^[^ ^27^ ^]^
FeSO_4_^.^·7H_2_O, FeCl_3_^.^ ·6H_2_O, NaOH	RCOOHor silane coupling agents	–	Crystalline spherical	–	–	50	110	^[^ ^282^ ^]^
FeCl_3_, FeCl_2_, H_3_PO_4_ (50% vol), H_2_SO_4_ (25% vol)	Polyvinyl chloride polymers	–	Crystalline spherical	–	–	8.7–10.2	70	^[^ ^283^ ^]^
Aqueous FeCl_3_, N_2_H_4_·H_2_O	Sodium bis(2‐ethylhexyl) sulfosuccinate	–	Spherical	D: 12–27	–	11	150	^[^ ^284^ ^]^
FeCl_3_^.^ ·6H_2_O, FeCl_2_^.^ ·4H_2_O	PEG, 3‐APTES	7.4	Spherical	–	–	13.3	80	^[^ ^285^ ^]^
FeCl_3_·6H_2_O, FeCl_2_·4H_2_O, HCl (37%)	PEG 4000	10	Crystalline spherical	14	–	12	90	^[^ ^281^ ^]^
FeCl_3_^.^ ·6H_2_O, FeCl_2_^.^ ·4H_2_O, NaOH, HCl, Trisodium citrate, Absolute ethanol, aq ammonia, tetraethyl orthosilicate	Amorphous silica	7.5–14	Crystalline spherical	–	–	50	–	^[^ ^286^ ^]^
FeCl_3_ · 6H_2_O, H_2_O_2_, NH_3_, diethylene glycol	Oxalic acid	–	Crystalline spherical	18.5	177 ± 42	–	200	^[^ ^287^ ^]^
FeCl_3_·6H_2_O, H_2_O_2_, NH_3_, diethylene glycol (DEG)	Formic acid	–	Crystalline spherical	15.8	117 ± 45	–	200	^[^ ^287^ ^]^
Fe_3_O_4_, H_2_O	Dextran	–	–	–	–	D:34.3	25	^[^ ^288^ ^]^
FeCl_3,_ FeCl_2_, HCl, NH_4_OH	(3‐Aminopropyl) trimethoxysilane	–	–	–	–	18.9	21–25	^[^ ^289^ ^]^
FeCl_2_·4H_2_O, Fe (NO_3_)_3_	EDTA‐Na2	–	Crystalline Spherical	D: 8.2	15	8	–	^[^ ^290^ ^]^
Fe_3_O_4_@SiO_2_	Glycine	–	Spherical	–	–	D: 57.24	90	^[^ ^291^ ^]^
Fe_3_O_4_@SiO_2_	Malonic acid	–	Spherical	–	–	D: 229.43	90	^[^ ^291^ ^]^
CH_3_CH_2_OH, 30% NH_3,_ Fe_3_O_4_	SiO_2_	–	Amorphous spherical and rod‐shaped	–	75.16 ± 15.75	53.20 ± 4.52	25	^[^ ^291^ ^]^
Fe_3_O_4_/epoxy nanocomposites.	3‐Aminopropyltriethoxy‐silane.	–	Crystalline spherical	14–17	14–20	–	70	^[^ ^292^ ^]^

## 8 Zinc Oxide (ZnO)

Zinc oxide nanoparticles (ZnO) are extensively researched metal oxide materials.^[^ ^180^ ^]^ ZnO's multifunctional qualities and the ability to modify its size and shape offer numerous choices for tuning its properties, which has generated significant attention. ZnO nanoparticles offer the benefit of effectively suppressing the activity of harmful microorganisms even at low concentrations.^[^ ^181^ ^,^ ^182^ ^]^ It is employed in diverse fields, including various,^[^ ^183^ ^]^ gas sensors,^[^ ^184^ ^]^ UV photodetector materials,^[^ ^185^ ^]^ high‐efficiency green phosphors,^[^ ^186^ ^]^ field emission displays,^[^ ^187^ ^]^ photovoltaic cells,^[^ ^188^ ^]^ beauty aids, and other demands. Different methods of synthesis can be utilized to generate ZnO nanoparticles and nanostructures, and the selection of methods can be impacted by the required characteristics, such as the sol–gel method,^[^ ^167^ ^]^ the hydrothermal technique,^[^ ^168^ ^]^ the precipitation method,^[^ ^169^ ^]^ and the chemical vapor deposition technique.^[^ ^170^ ^]^ To improve ZnO nanocrystals’ properties in various applications, their surface must be modified. Research performed by Wu et al. explored the modification of ZnO nanocrystals by using various categories of organic capping agents. This manipulation is to control the particle growth, stabilize it, and improve its optical properties. Colloidal synthesis using an alcoholic solution of zinc acetate dihydrate at 68 °C is carried out under ambient conditions. The successful limitation of ZnO nanoparticle size for average sample sizes within the 10–20 nm range with agglomeration was achieved by the additional capping action of 3‐aminopropyl trimethoxysilane, tetraethyl orthosilicate, and mercapto succinic acid.^[^ ^180^ ^]^ Zinc oxide nanoparticles were functionalized with polymethacrylic acid (PMAA) to enhance their aqueous dispersion. TGA is used to quantify the amount of PMAA coating and assess the modified nanoparticles’ thermal stability. XRD demonstrated the material's crystallinity. The appearance of PMAA causes the size to decrease of the crystallite. TEM analysis of morphology showed that PMAA coating improved dispersion and prevented aggregation.^[^ ^189^ ^]^ Covering ZnO with oleic acid, the hydroxylic groups on the surface of ZnO nanoparticles interact chemically with organic long‐chain molecules to form a covalent connection. During the process, the following reaction occurred:

(12) $$\mathrm{ZnO}{\left(\mathrm{OH}\right)}_{x}+y\mathrm{HOOC}{\left({\mathrm{CH}}_{2}\right)}_{7}\mathrm{CH}\mathrm{CH}{\left({\mathrm{CH}}_{2}\right)}_{7}{\mathrm{CH}}_{3}\to \mathrm{ZnO}{\left(\mathrm{OH}\right)}_{x}-y{\left[\mathrm{OOC}{\left({\mathrm{CH}}_{2}\right)}_{7}\mathrm{CHCH}{\left({\mathrm{CH}}_{2}\right)}_{7}{\mathrm{CH}}_{3}\right]}_{y}+yH_{2}O$$

To produce ZnO nanoparticles, zinc acetate and ammonium carbonate precipitate were calcined for three hours at 450 °C. The wurtzite hexagonal structure of ZnO is responsible for the different peaks shown in the X‐ray diffraction (XRD) study. The peaks had the same properties, but they were oriented differently, suggesting that they matched the same nanoparticles’ diffraction pattern. Transmission electron microscopy (TEM) was used to analyze the ZnO nanoparticles’ size and form. It was confirmed that the particles maintained their spherical shape, with a common size of 20–30 nm. The SEM pictures demonstrate that the shape of the ZnO nanoparticles treated with oleic acid is similar to that of the unmodified ZnO nanoparticles.^[^ ^172^ ^]^ Following 8 min of gas phase treatment with propiolic acid at a pressure of 1 Torr, the structure of the 100% zinc oxide powder is completely maintained. If propiolic acid is employed in the liquid phase for processing, it significantly disrupts the organization of the nanopowder. SEM is used to analyze both of these structures.^[^ ^173^ ^]^ The addition of γ‐aminopropyltriethoxysilane (KH550) changed the surface of ZnO, which brought in organic functional groups. The hexagonal wurtzite structure was preserved following alteration, as demonstrated by the XRD examination of the modified ZnO nanoparticles, which revealed a crystalline diameter in the 20–28 nm range. The typical particle size distribution of the ZnO particles, according to TEM, is between 30 and 50 nm.^[^ ^174^ ^]^ Concanavalin A (Con A)‐coated zinc oxide nanoparticles (ZnO‐NP) were utilized to immobilize β‐galactosidase on a bio‐affinity platform. The TEM investigation revealed that the produced zinc oxide nanoparticles were 28.5 nm in size.^[^ ^190^ ^]^ Khurana et al. conducted research where vinyltriethoxysilane (VTES) was added to modify zinc oxide nanoparticles at different quantities. ZnO nanoparticles treated with VTES showed strong peaks that were almost the same as those of unmodified zinc oxide nanoparticles, according to the researchers. Accordingly, the crystalline structure of the ZnO nanoparticles was unchanged. The SEM study revealed that the ZnO nanoparticles underwent surface modification using VTES, which effectively prevented their aggregation. A decrease in agglomeration of ZnO nanoparticles commences with a VTES concentration of 2.5 wt%.^[^ ^191^ ^]^ Zinc oxide (ZnO) was subjected to treatment with stearic acid (SA) in a 50/50 weight ratio. This mixture was subsequently included in the high‐density polyethylene (HDPE) matrix. The operating circumstances included a rotational speed of 50 revolutions per minute and a temperature of 200° Celsius. The SEM micrographs demonstrate the transformation of ZnO particles from an inorganic state to an organic one, resulting in a decrease in their surface tension and an improvement in their dispersion within the PE matrix.^[^ ^192^ ^]^ Tang et al. modified the ZnO nanoparticles’ surface using methacryloxypropyltrimethoxysilane (MPS). ZnO nanoparticles were produced using a uniform precipitation method. ZnO nanocrystals were created by calcining the ZnO precursor powder at 350 °C. The XRD result shows that the spectra of the MPS‐modified ZnO nanoparticles and the untreated ZnO nanoparticles are almost the same.^[^ ^193^ ^]^ Various mercaptoacetic acid (MAA) addition times were used to modify ZnO nanorods in the research conducted by Song et al. The SEM result showed that after adding MAA for 1 h, the samples’ morphology seemed disorganized. The majority of the samples have helical shapes, with very few having rod‐shaped features. The ZnO nanorods showed a disordered structure and a nubby‐like morphology with the addition of MAA at the 3‐h mark. The SEM images showed the presence of two different morphologies when MAA was introduced at 5 h. There is a rod‐shaped structure and a column‐shaped structure.^[^ ^194^ ^]^ Using polyvinylpyrrolidone (PVP), Wurtzite ZnO nanocrystals were created. The TEM images demonstrate that the PVP‐capped nanoparticles are uniformly distributed and do not exhibit any aggregation, but the noncapped ZnO nanoparticles tend to aggregate. The size distribution of the ZnO nanoparticles that were modified with PVP is noticeably smaller than that of the nanoparticles that were not. The PVP‐modified ZnO nanoparticles exhibit a spherical shape, whereas the unaltered ZnO nanoparticles have an ellipsoid shape.^[^ ^195^ ^]^ Ma et al. modified ZnO with KH570 (gamma‐methacryloxypropyltrimethoxysilane). The ZnO and KH570 mixture was constantly shaken for an hour at a rate of 5000 r s^−1^. The completed products were vacuum‐dried for 12 h at 80 °C. Soxhlet extraction was ultimately used to acquire the material. The ZnO nanoparticles were observed to agglomerate to some degree, according to TEM images; however, the KH570 grafted ZnO nanoparticles showed significantly improved dispersion and size regularity.^[^ ^196^ ^]^ Agrawal et al. did a study in which they treated ZnO using palmitic acid. The ZnO nanoparticles were produced using a hydrothermal technique. The presence of diffraction peaks provides evidence supporting the wurtzite hexagonal structure of the ZnO nanoparticles. The size of the crystalline was determined to be 24 nm. The TEM image of the PA‐ZnO material confirms that the surface of ZnO is fully grafted by palmitic acid. The SEM image verifies that the PA‐ZnO sample exhibits a rough surface on top of the substrate, with spaces of air trapped among the nanoparticles that are distributed randomly.^[^ ^197^ ^]^ Punnoose et al. modified ZnO with two samples. Diethylene glycol was employed as a medium for operation and zinc acetate dehydrate as a precursor in one of his hydrolysis technique tests. For 90 min, the solution was kept at 150 °C. Using denatured ethanol as a reaction liquid and zinc acetate dihydrate as a precursor, the second sample was made by a wet chemical method. For 90 min, the solution was kept at 80 °C. X‐ray diffraction shows that the average crystallite sizes of these ZnO NP samples are similar, measuring 9.38 nm for the first sample and 9.15 nm for the second.^[^ ^198^ ^]^ Shahmoradi et al. modified ZnO using n‐butylamine and caprylic acid as structural modifiers under mild hydrothermal conditions. The powder XRD data demonstrated highly crystallized wurtzite and anatase structures. The modification with caprylic acid resulted in a change in the crystal structure. The SEM images demonstrate that surface modification can alter the modified ZnO nanoparticles’ size, shape, and dispersibility.^[^ ^199^ ^]^ Nanoscaled ZnS crystals have garnered increasing interest lately due to their size and shape‐dependent photoemission properties and quantum confinement effect. In order to study properties that rely on size and shape, a lot of work has gone into regulating the size and form of ZnS nanostructures. The precursor can undergo thermal breakdown at low temperatures to produce wurtzite ZnS nanorods.^[^ ^200^ ^]^ Mazloom et al. used an anionic sodium dodecyl sulfate (SDS) surfactant as a size and morphology controller to create zinc vanadate (Zn_3_V_2_O_8_) nanostructures using a straightforward precipitation technique.^[^ ^201^ ^]^ Relevant data are registered in **Table** **3**.

**Table 3:** Surface modification data of ZnO nanoparticles via different organic modifiers.

Reagent	Modifier	pH	Morphological structure	XRD	SEM	TEM	Calcination Temp. [°C]	References
				[nm]				
	Polymethacrylic acid (PMAA)	5.0–5.5	Crystalline	–	–	100	–	^[^ ^189^ ^]^
	Aminopropyltriethoxysilane	–	Crystalline, spherical, or hexagonal		25–45	20	800	^[^ ^293^ ^]^
	vinyl triethoxysilane	–	–	19.58 to 20.57	20	–	–	^[^ ^191^ ^]^
Aqueous solutions of Zn(CH_3_COO)_2_·2H2O and (NH4)_2_CO_3_	Polystyrene	–	Crystalline quasi‐spherical	20	–	20	450	^[^ ^229^ ^]^
	γ‐Aminopropyltriethoxysilane	–	Crystalline quasi‐spherical	24–37	Below 60	30–50	–	^[^ ^294^ ^]^
Zinc acetate dihydrate	3‐Aminopropyl trimethoxysilane, tetraethyl orthosilicate, mercaptosuccinic acid, 3‐mercaptopropyl trimethoxysilane and polyvinylpyrrolidone	–	Crystalline spherical	–	10–30	6 to 10	–	^[^ ^180^ ^]^
	KH570	–	Crystalline	–	–	20	–	^[^ ^196^ ^]^
	Caprylic acid and n‐butylamine.	–	Crystalline small prism‐like	5.2–7.8	–	–	–	^[^ ^199^ ^]^
Zn acetate dehydrate	Diethylene glycol	–	Crystalline	9.15–9.38	–	–	800	^[^ ^198^ ^]^
Zinc acetate dihydrate	3‐(Trimethoxysilyl)propylmethacrylate		Crystalline	5.24, 4.34 and 4.19		4.02 ± 0.21, 3.86 ± 0.09, 3.85 ± 0.1		^[^ ^295^ ^]^

## 9 Aluminum Oxide (Al_2_O_3_)

Aluminum oxide nanoparticles (AlNPs) are porous components that fall under the category of metal oxide nanomaterials. Structurally, they consist of a corundum‐like arrangement where six oxygen atoms surround one aluminum atom. AlNPs are inexpensive, readily available, and easy‐to‐handle nanoparticles with a large surface area, mechanical force, and exceptional chemical stability. However, they exhibit low electrical conductivity. Furthermore, the remarkable optical characteristics of AlNPs are employed as a prototype for studying the properties, structural modifications, and electrical changes of nanomaterials.^[^ ^202^, ^203^, ^204^, ^205^, ^206^, ^207^, ^208^ ^]^ AlNPs can be synthesized using various techniques employing straightforward and cost‐efficient processes.^[^ ^209^ ^]^ Their synthesis methods can be categorized as solid phase‐based,^[^ ^210^ ^]^ gas phase‐based,^[^ ^211^ ^]^ and liquid‐based,^[^ ^212^ ^]^ procedures. These methods include mechanical ball milling,^[^ ^213^ ^]^ mechanochemical processes,^[^ ^214^ ^]^ solution reduction,^[^ ^215^ ^]^ exploding wire techniques,^[^ ^216^ ^]^ decomposition,^[^ ^217^ ^]^ and gas evaporation.^[^ ^218^ ^]^ It is used for drug delivery, cancer therapy, antimicrobial activity, and antimicrobial impacts, treatment of other diseases, biomolecular preservation, and immunotherapy.^[^ ^219^ ^]^ Extensive research has focused on modifying Al_2_O_3_ NP surfaces with organic groups to improve their crystallization behavior at low concentrations.^[^ ^220^ ^,^ ^221^ ^]^ Jiang et al. investigated the mechanical characteristics and crystallization dynamics of iPP/modified‐Al_2_O_3_ nanocomposites. The Al_2_O_3_ NPs were modified with benzoic acid to alter their surface chemistry. Alumina nanoparticles were refluxed overnight in toluene with benzoic acid to obtain functionalized particles. These particles were then centrifuged and washed with 2‐propanol and ethanol to eliminate unreacted acid. The resulting BA‐Al_2_O_3_ nanoparticles were next oven‐dried at 80 °C. Without changing the shape of their crystals, benzoic acid functionalization enhanced the alumina nanoparticles’ mechanical characteristics and aggregation tendency. The covalent bonding of benzene rings to the nanoparticle surface suggests enhanced polymer‐nanoparticle interactions.^[^ ^222^ ^]^ Alumina has several crystal forms, including θ, δ, γ, η, and δ phases, etc.^[^ ^223^ ^]^ The aluminum crystal structures change and eventually turn into alpha‐Al_2_O_3_ when the temperature rises above their melting point.^[^ ^195^ ^]^ Since α‐Al_2_O_3_ is usually produced at a calcination temperature above 1000 °C, obtaining nano α‐Al_2_O_3_ powder at high calcination temperatures is challenging. A considerable percentage of α‐Al_2_O_3_ particle coarsening and clumping is unavoidably caused by an elevated calcination temperature.^[^ ^196^ ^]^ Triethanolamine (TEA) was used as the chelate agent for the first time to address this issue. Crystal growth may be accelerated, decelerated, or inhibited by chelate agents. After calcining, the poly‐esterification network that was created by triethanolamine and citric acid yielded nanoparticles.^[^ ^195^ ^]^ Ascorbic acid and citric acid can chelate with NPs, making them effective and environmentally friendly modifiers.^[^ ^224^ ^,^ ^225^ ^]^ In their study, Mallakpour et al. used ascorbic acid and citric acid to modify Al_2_O_3_. The altered and pure Al_2_O_3_ FE‐SEM images demonstrated how the modifiers applied to their surfaces changed the surface structure of the material. The modified Al_2_O_3_ NPs were uniformly distributed throughout the polymer matrix. This dispersion creation may confirm strong interfacial connections, such as van der Waals and hydrogen, between the substrate and filler.^[^ ^199^ ^]^ General modification reaction by the citric acid & ascorbic acid can be represented as follows:

(13) $${\mathrm{Al}}_{2\;}O_{3}\left(\mathrm{unmodified}\right)+{2C}_{6\;}H_{8\;}O_{7}\;\left(\mathrm{citric}\mathrm{acid}\right)+{\mathrm{Al}}_{2}\;O_{3}\to {\mathrm{modified}\;\mathrm{Al}}_{2\;}O_{3}+{3H}_{2}\;O$$

(14) $${\mathrm{Al}}_{2\;}O_{3}\left(\mathrm{unmodified}\right)+{6C}_{6\;}H_{7}O_{6}\left(\mathrm{ascorbic}\mathrm{acid}\right)+{\mathrm{Al}}_{2}\;O_{3}\to {\mathrm{modified}\;\mathrm{Al}}_{2\;}O_{3}+{3H}_{2}\;O$$

Brandon et al.'s study found that 1,2‐epoxy‐9‐decene‐capped air‐stable Al NPs with a 20–25 nm particle size were produced. Materials with limited air stability were formed by both the 1:1 and 20:1 Al: epoxide mole ratios, suggesting that the Al: capping agent mole ratios under investigation had an impact on nanoparticle stability but no discernible impacts on particle size or particle size distribution. Epoxides have demonstrated efficiency as capping agents, producing Al nanostructures with well‐controlled sizes and high concentrations of active Al. Al NPs are encased in a hydrophobic polymer matrix as a result of the polymerization of the epoxide's terminal alkene functionality. Furthermore, by employing a cross‐linker known as 1,13‐tetradecadiene to encourage the polymerization of the final alkene, we have produced materials that are even more resistant to alterations over time and exhibit minimal degradation during six months of exposure to air.^[^ ^226^ ^]^ The surface characteristics of aluminum oxide nanoparticles were modified using three silanes having alkyl groups: octadecyltrimethoxysilane (C18), methyltrimethoxysilane (C1), and octyltriethoxysilane (C8).^[^ ^227^ ^]^ Scanning electron microscopy was used to evaluate the coated nanoparticles’ dispersion within the low‐density polyethylene after they were mixed with the polymer. By applying incremental strain to the nanocomposites and examining the strained composite materials under a scanning electron microscope, the interfacial adhesion between the nanoparticles and matrix was assessed. For 24 h, aluminum oxide nanoparticles were vacuum‐dried at 190 °C. These nanoparticles were then dispersed in a water/2‐propanol solution and sonicated for 5 min to create a homogeneous suspension.^[^ ^228^ ^]^ Ammonia (25 wt%) was added to the suspension under vigorous stirring, followed by silane addition and a reaction time of t hours at room temperature. Different silanes (C1, C8, and C18) were applied to the surface of alumina nanoparticles to enhance their interfacial adhesion and dispersion in LDPE. While C18‐coated nanoparticles demonstrated the maximum strain for cavitation, indicating the strongest interfacial adhesion, C8‐coated nanoparticles demonstrated the best dispersion. To increase adhesion in Al_2_O_3_/polymer laminates, the silane coupling agent TEVS was added to the alumina surface. Because of the enhanced interfacial adhesion, the three‐point bending tests did not result in catastrophic failure, and the crack deflection at the weak interfaces greatly increased toughness.^[^ ^229^ ^]^ More examples on the structural modification on Al_2_O_3_ is given in **Table** **4**.

**Table 4:** Surface modification data of Al_2_O_3_ nanoparticles via different organic modifiers.

Reagent	Modifier	pH	Morphological structure	XRD	SEM	TEM	Calcination Temp. [°C]	References
				[nm]				
Al_2_O_3_ NPs powder, o‐xylene	Oleic acid	–	Crystalline quasi‐spherical	–	30 × 10^3^	–	50	^[^ ^296^ ^]^
Al_2_O_3_ NPs powder, Sodium silicate solution, H_2_SO_4_	SiO_2_	9.5	Crystalline quasi‐spherical	–	30 × 10^3^	–	85–90	^[^ ^296^ ^]^
PVC, tetrahydrofuran, nanosized *α*Al_2_O_3_ powder	Citric acid & ascorbic acid	–	Crystalline spherical network	–	–	25–50	400	^[^ ^297^ ^]^
Nanosized a‐Al_2_O_3_ powder, PVP	Citric acid & ascorbic acid	–	Crystalline spherical	–	–	12	25	^[^ ^298^ ^]^
N, N‐Dimethylethylamine alane, titanium (IV) isopropoxide, 1,2,7,8‐diepoxyoctane	1,2‐epoxy‐9‐decene	–	Crystalline spherical	12–24		20–25	25	^[^ ^299^ ^]^
Aluminum nitrate nonahydrate	Citric acid, TEA	–	Crystalline semi‐ spherical	29	>200	–	80	^[^ ^299^ ^]^
Aluminum nitrate nonahydrate, Cu(NO_3_)_2_ ·3H_2_O	Urea	–	Crystalline plate‐like	–	–	10–20	250	^[^ ^300^ ^]^
LDPE, aluminum oxide Nps, ethanol, ammonia, 2‐propanol	Methyltrimethoxysilane	–	Crystalline spherical	–	–	2.5	25	^[^ ^227^ ^]^
Al_2_O_3_ NPs, epoxy resin, xylene, and butane mixture	4‐Aminobutyltriethoxysilane	–	Crystalline spherical to spheroidal	26	–	26–32	30–120	^[^ ^301^ ^]^
*α*‐Al_2_O_3_ NPs, water, ethanol solution, NaOH	Citric acid	–	Crystalline spherical	–	18	15	15	^[^ ^302^ ^]^

## 10 Copper Oxide (CuO)

One prominent transition metal oxide that is well‐known for its exceptional properties is copper (II) oxide (CuO), including strong resistance to heat, exceptional ability to conduct electricity, and intrinsic catalytic qualities.^[^ ^230^ ^]^ Copper oxide (CuO) has drawn significant interest due to its antibacterial and biocidal attributes, making it suitable for various biomedical functions.^[^ ^231^ ^]^ CuO is an important p‐type semiconductor that has been utilized in several applications, including gas sensors,^[^ ^232^ ^]^ catalytic processes,^[^ ^233^ ^]^ accumulators,^[^ ^234^ ^]^ high‐temperature superconductors,^[^ ^235^ ^]^ solar energy conversion,^[^ ^236^ ^]^ field emission emitters.^[^ ^237^ ^]^ CuO NPs are being examined for various functions, including antibacterial agents in fabrics and healthcare, wood preservatives made of nano‐ and micro‐sized CuO particles, and ink modifiers made of single Cu NPs or in conjunction with additional metallic NPs.^[^ ^238^, ^239^, ^240^ ^]^ In a study by Solaimeni et al. palmitic acid and stearic acid altered the surfaces of CuO nanoparticles. By mixing CuCl_2_·2H_2_O with ammonia solution and calcining the resulting product for five hours at 450 °C, CuO nanoparticles (NPs) were produced chemically. The reaction equation is as follows:

(15) $${\mathrm{CuCl}}_{2}\cdot {2H}_{2}O+{2\mathrm{NH}}_{4}\mathrm{OH}\to \mathrm{Cu}{\left(\mathrm{OH}\right)}_{2}+{2\mathrm{NH}}_{4}\mathrm{Cl}+{2H}_{2}O$$

(16) $$\mathrm{Cu}{\left(\mathrm{OH}\right)}_{2}\left(\mathrm{calcined}\;450\;{}^{\circ}C,5h\right)\to \mathrm{CuO}+H_{2}O$$

The XRD data show the same pattern between modified and unmodified CuO. The SEM images show that Uncoated copper oxide nanoparticles exhibit significant agglomeration. The dispersion of particles was marginally enhanced when their surface was changed with stearic acid, resulting in a modest increase in particle size.^[^ ^46^ ^]^ EDTA‐capped copper oxide's XRD peak location matched that of EDTA‐capped CuO, and the material's crystalline nature was indicated by the strong, highly concentrated peaks. The SEM images show that uncapped CuO nanoparticles are roughly spherical. Nevertheless, the structure of the particles could not be clarified by EDTA‐capped CuO nanoparticles. In comparison to uncapped nanoparticles, the SEM pictures also demonstrate how strongly agglomerated capped nanoparticles are. In this study, ascorbic acid and citric acid were used to modify CuO. After being treated with CA and AA, the CuO NPs show almost identical XRD patterns. This implies that following the surface modification, the CuO NPs’ crystalline structure stays the same. The altered copper oxide nanoparticles are uniformly distributed inside the polymer matrix, as seen by the FESEM photos, which reduces aggregation. The NC 12 weight percent's FESEM image shows minimal agglomeration.^[^ ^241^ ^]^ On the surface of CuO nanoparticles, 2,3,4,5‐tetrabromo‐6‐[(4‐hydroxyphenyl) carbamoyl] benzoic acid was used as a new coupling agent to enhance their dispersion. The XRD pictures show that after surface modification, the crystalline structure of the modified CuO did not change. SEM images showed that these NPs were spherical and had a size of 29 nm. Because of their high surface energy, the CuO nanoparticles are seen to cluster in specific locations.^[^ ^242^ ^]^ In the modification of CuO with oleic acid, CuO nanoparticles were synthesized using the chemical precipitation approach by reacting Cu(NO_3_)_2_ · 3H_2_O with a solution of caustic soda. The solid was thereafter subjected to calcination at 400 °C for 4 h. According to XRD diffraction, the modified CuO NPs have a wavelength of 60 nm. SEM photos demonstrate that the size of the changed CuO by oleic acid is 56 nm. After being exposed to oleic acid, the CuO nanoparticles are composed of irregularly arranged and scattered particles.^[^ ^243^ ^]^ The green modifier polyvinyl alcohol (PVA) was grafted onto the surface of CuO nanoparticles (NPs) to create the CuO‐PVA nanohybrid. The modified CuO nanoparticles’ spectra closely mirror the unmodified particles’ spectra based on XRD measurements. The crystalline structure of the CuO NPs was unaffected by the surface alteration. After applying a PVA coating, the average particle size of CuO NPs decreased from 30 to 15 nm, as seen by the TEM pictures.^[^ ^222^ ^]^ Oleylamine (OLA) was used by Ranjith Kumar et al. to cap CuO. The peaks found in the sample's XRD pattern are the same as those of monoclinic CuO. According to the HRTEM image, the OLA‐CuO nanoparticles had a spherical shape distribution with an average size of 4 ± 0.3 nm. Additionally, it demonstrates that polyamine inhibits nanoparticle agglomeration.^[^ ^223^ ^]^ Chiral diacids (DAs), which are made of amino acids, were used to modify the surface of CuO nanoparticles (NPs) to improve their dispersibility. The surface modification approach was created using ultrasonic irradiation. An X‐ray diffraction study shows that even after surface modification with DA, the crystalline structure of copper oxide nanoparticles remains unchanged. According to the FE‐SEM pictures, the nanoparticles were uniformly distributed and had a low propensity to aggregate. Compared to normal CuO NPs, the CuO nanoparticles (NPs) treated with bioactive DAs showed a smaller range of sizes.^[^ ^224^ ^]^ Variation on the structure of the CuO is provided in **Table** **5**.

**Table 5:** Surface modification data of CuO nanoparticles via different organic modifiers.

Reagent	Modifier	pH	Morphological structure	XRD	SEM	TEM	Calcination Temp. [°C]	References
				[nm]				
[Cu(CH_3_CO)_2_^.^H2O], Urea, Ethanol	EDTA	–	–	3–8	–	–	130	^[^ ^303^ ^]^
CuO NPs, castor oil, ethanol, paraffin oil	Sodium dodecyl sulfate	–	Regular spherical	–	80	–	70	^[^ ^304^ ^]^
Cupric nitrate solution, aq. NaOH solution	Polyethylene glycol (PEG)	–	Crystalline rectangular shape	23.30	–	504.4	140	^[^ ^305^ ^]^
Cupric nitrate solution, aqueous NaOH solution	Polyvinylpyrrolidone (PVP)	–	Crystalline quasi‐rectangular shape	22.86	–	417.9	140	^[^ ^305^ ^]^
Cupric nitrate solution, aqueous NaOH solution	Polyvinyl alcohol (PVA)	–	Crystalline brick‐like	21.79	–	266.5	140	^[^ ^305^ ^]^
Cupric nitrate solution, aqueous NaOH solution	Polydopamine (PDA)	7.4	Crystalline rod‐like	24.03	–	87.7	140	^[^ ^305^ ^]^
CuO nanopowder, ethanol, water	2,3,4,5‐tetrabromo‐6‐[(4‐hydroxyphenyl) carbamoyl]	–	Crystalline spherical	14	–	27 ± 4	25	^[^ ^242^ ^]^
CuO NPs	Citric acid & vitamin C	–	–	–	–	10.80 ± 2.91	–	^[^ ^306^ ^]^
CuO, NaOH	Tryptophan	–	Crystalline spherical	–	–	≈ 30	80–90	^[^ ^307^ ^]^
CuO NPs, NaOH	Sodium oleate	–	Crystalline spherical	–	–	D: 20	90	^[^ ^308^ ^]^

## 11 Nickel Oxide (NiO)

NiO is one of the major transition metal oxide materials that possesses a cubic lattice formation. The nickel oxide nanoparticles possess distinctive characteristics, including optical, thermal, electrical, chemical, and physical properties. The material demonstrates anodic electrochromism, exceptional longevity and electrochemical stability, significant spin optical density, and diverse manufacturing capabilities. The following are a few noteworthy applications of NiO: Energy Storage and Conversion,^[^ ^244^ ^]^ catalysis,^[^ ^245^ ^]^ magnetic,^[^ ^246^ ^]^ Optoelectronics,^[^ ^247^ ^]^ Biomedical Applications,^[^ ^248^ ^]^ Environmental Application,^[^ ^249^ ^]^ solar cells,^[^ ^250^ ^]^ and many more. Nickel oxide (NiO) nanoparticles can be synthesized using various methods that include the sol–gel method,^[^ ^251^ ^]^ hydrothermal method,^[^ ^252^ ^]^ precipitation method,^[^ ^253^ ^]^ thermal decomposition,^[^ ^254^ ^]^ microemulsion method,^[^ ^255^ ^]^ combustion,^[^ ^256^ ^]^ green synthesis,^[^ ^257^ ^]^ each with unique benefits and difficulties. Wang et al. conducted a study where they treated NiO with PPA (phenyl phosphoric acid), PPA‐NH_2_, and PPA‐Br for improving the photocatalytic activity of NiO toward hydrogen generation. The crystalline structure of NiO remained unchanged following alteration, according to the XRD data. The SEM scans indicated that the morphology of NiO is not dramatically affected. HRTEM research suggests that the addition of organic phosphonic acid does not modify the original structure of NiO, which consists predominantly of irregular nanoparticles with a typical size of roughly 50 nm.^[^ ^258^ ^]^ Soleimani and Niavarzi worked with PMMA/NiO polymer nanocomposites. In this work, he modified NiO with oleic acid. NiO nanoparticles (NPs) were synthesized when they reacted with NiCl_2_ · 6H_2_O along with caustic soda, then calcinated the resulting precipitate at 300 °C for 2 h. The reaction can be illustrated as follows:

(17) $${\mathrm{NiCl}}_{2}\cdot {6H}_{2}O+2\mathrm{NaOH}\to \mathrm{Ni}{\left(\mathrm{OH}\right)}_{2}+2\mathrm{NaCl}+{6H}_{2}O$$

(18) $$\mathrm{Ni}{\left(\mathrm{OH}\right)}_{2}\left(\mathrm{calcined}\;300\;{}^{\circ}C,2h\right)\to \mathrm{NiO}+H_{2}O$$

The XRD data indicate that the peak properties remain unchanged following surface modification, and are identical to those of the unmodified NiO NPs, which possess cubic crystal structures having a face‐centered orientation. In SEM images, NiO particles are typically 38 nm in size, but when exposed to oleic acid, their average size changes to 43 nm.^[^ ^259^ ^]^ When NiO nanoparticles are treated with stearic acid, their morphology does not alter, based on TEM pictures of the modified and unmodified NiO nanoparticles. 14.4 ± 2.7 and 13.2 ± 1.6 nm are the average sizes of unmodified and modified NiO nanoparticles, respectively.^[^ ^260^ ^]^ Chen et al. worked on NiO films, where they used Nickel (II) acetate tetrahydrate as the precursor. Two different kinds of solvents were used in the experiment. Among them was an alcoholic. The films made from alcoholic solutions showed broad, weak reflections in their XRD patterns, indicating that they were X‐ray amorphous. Talebian et al. used nickel nitrate as a precursor to create NiO using the sol–gel process. It was subsequently treated with solvents such as methanol, 1,4‐butanediol, ethanol, and 2‐propanol, which have polarities and viscosities that differ greatly. The NiO films show no indications of particle aggregation, according to the SEM pictures. The films consist of consistently sized particles, ranging from 38 to 80 nm. The NiO‐B film exhibits enhanced crystallinity and a very permeable structure. The TEM picture of the film reveals a granular structure in the NiO films, characterized by a reasonably uniform size distribution.^[^ ^261^ ^]^ It was examined how NiO nanoparticles affected the adsorption of Q‐65. A regulated thermal dehydroxylation process of Ni(OH)_2_ was used to produce NiO nanoparticles with diameters varying from 5 to 80 nm. The broad peaks from XRD suggest that the produced nanoparticles have a significantly small crystallographic domain size. According to the HRTEM images, the nanoparticle particles, with sizes of 20 and 30 nm, appear to be composed of thin, flat sheets that are joined together. The samples at 40 and 60 nm appear to be cylindrical objects, single thicker sheets, and hexagonal shapes, respectively.^[^ ^262^ ^]^ NiO nanosheets were modified with polyaniline, where NiO was synthesized from nickel sulfate precursor. The alteration of the NiO film was conducted through in situ polymerization in a polyaniline solution. The SEM images show that the morphology of the NiO film has altered significantly. The XRD results show the similarity between NiO and modified NiO.^[^ ^263^ ^]^ Yang Bai et al. modified NiO nanocrystal layer surfaces using diethanolamine on perovskite crystallization. The XRD images showed that the DEA alteration did not alter the growth orientation of perovskite crystals on the NiO nanocrystal layer. The SEM images show that a substantial quantity of pinholes is distinctly observed in the perovskite layer on the uncoated NiO film. Still, the quantity of pinholes is markedly diminished following DEA treatment.^[^ ^264^ ^]^ NiO prepared from Ni(NO_3_)_2_⋅·6H_2_O precursor was modified using oleic acid and later grafted with polystyrene. The preparation reaction is given below:

(19) $$\mathrm{Ni}{\left({\mathrm{NO}}_{3}\right)}_{2}\cdot {6H}_{2}O+\mathrm{NaOH}+\mathrm{NaOCl}\to {\mathrm{NiO}}_{2}↓+{2\mathrm{NaNO}}_{3}+\mathrm{HCl}+{6H}_{2}O$$

(20) $${\mathrm{NiO}}_{2}+{\mathrm{CH}}_{3}{\mathrm{CH}}_{2}\mathrm{OH}\to \mathrm{NiO}+{\mathrm{CH}}_{3}\mathrm{CHO}+H_{2}O$$

The XRD results indicate that the incorporation of embedded polystyrene into NiO nanoparticles reduces the crystallinity of the nanoparticles, resulting in a decrease in both the height and intensity of the peaks in the nanocomposites. The SEM images show the improvement in the dispersion of modified NPs, which are non‐uniform. The average dimension of the particles for both is roughly 16 nm. It also showed that PSt/NiO nanocomposites are entirely spherical particles resembling dandelion flowers.^[^ ^265^ ^]^ With the presence of Disodium Palmitic acid, the NiO produced was small and monodispersed. Ni(NO_3_)_2_ · 6H_2_O as a precursor and ethanol as solvent, the NiO produced was monetized with and without Na_2_PA. The NiO produced without the presence of Na_2_PA shows a greater size. XRD images show more crystallinity. Without Na_2_PA, the shape of NiO shows a variety of spherical, cubic, and pentagonal shapes, which are 23–284 nm in size. Based on the TEM pictures, the average NiO‐NP particle size made with Na_2_PA was 19.1 ± 3.2 nm.^[^ ^266^ ^]^ Few recent works on the structural modification of NiO are registered in **Table** **6**.

**Table 6:** Surface modification data of NiO nanoparticles via different organic modifiers.

Reagent	Modifier	pH	Morphological structure	XRD	SEM	TEM	Calcination Temp. [°C]	References
				[nm]				
	Phenyl phosphoric acid, PPA‐NH_2_, PPA‐Br	–	Crystalline	–	–	50	60	^[^ ^258^ ^]^
NiCl_2_ · 6H_2_O	Oleic acid	–	Crystalline	–	43	–	300	^[^ ^259^ ^]^
Nickel acetate	Stearic acid	–	Crystalline	–	–	13.2±1.6	500	^[^ ^260^ ^]^
Nickel (II) carbonate	Acetone	–	Crystalline spherical	–	–	3–15	–	^[^ ^309^ ^]^
Ni(NO_3_)_6_ · 6H_2_O	methanol, 1,4‐butanediol, ethanol, and 2‐propanol	–	Crystalline and porous spherical	41,87,56,70	35–72	35–72	500	^[^ ^261^ ^]^
(Ni(NO_3_)_2_ · 6H_2_O)	citric acid	–	Cubic spherical	25	20 to 30	20	400	^[^ ^310^ ^]^
Ni(OH)_2_	Quinolin‐65	–	Crystalline rod‐like, thin sheet‐like, cylinder‐like, thick sheet‐like, hexagonal	5 to 80	5–80	5–80	–	^[^ ^262^ ^]^
NiSO_4_ · 6H_2_O	Dimethylglyoxime in ethanol	–	Poly‐crystalline rod‐like	17.6	–	18	400	^[^ ^254^ ^]^

## 12 Effective Parameters that Increase the Yield of Reactions

Certain key factors significantly influence the yield and performance of metal oxide nanoparticles. Choosing organic modifiers is important. Functional groups like carboxyl, amine, or hydroxyl help prevent the particles from clumping together and affect the development of specific shapes. For instance, citric acid resulted in smaller, evenly distributed TiO_2_,^[^ ^267^ ^]^ while EDTA led to well‐dispersed 15 nm Fe_3_O_4_.^[^ ^268^ ^]^ The pH of the medium is crucial because it affects hydrolysis and condensation. TiO_2_ treated with acetic acid at pH 3.4–3.9 formed monodispersed crystalline rods.^[^ ^269^ ^]^ In contrast, ZnO stabilized at pH 5–5.5 with silane showed better stability.^[^ ^189^ ^]^ It is necessary to adjust the concentrations of precursors and modifiers. Low concentrations can lead to inadequate surface coverage, while high concentrations can cause clumping. Regarding the formation of Fe_3_O_4_, a 5 wt% concentration of polyacrylic acid led to a uniform dispersion, whereas a 10 wt% concentration resulted in aggregation.^[^ ^270^ ^]^ The reaction temperature and calcination treatment also significantly influence the crystallinity, phase, and morphology. The TiO_2_ calcined in the range of 400–650 °C controlled anatase and rutile phases,^[^^]^ and the CuO calcined at 450 °C yielded homogenous crystalline particles.^[^ ^46^ ^]^ In addition, reaction times and stirring speeds have a significant effect on the complete nucleation and growth. Fe_3_O_4_ modified in the presence of PVP with stirring of 300–600 rpm resulted in controlled particle size.^[^ ^270^ ^]^ TiO_2_ coated with polydopamine exhibited better surface uniformity after 2 h of stirring.^[^ ^271^ ^]^ The type of solvent affects solubility and diffusion. Alcohol‐based solvents create uniform porous structures of NiO,^[^ ^261^ ^]^ and ZnO spheres that measure 20–30 nm.^[^ ^181^ ^]^ A key idea is the balance between nucleation and growth. When rapid nucleation is followed by controlled growth, it leads to monodisperse nanoparticles. This is seen in PVP‐capped ZnO,^[^ ^195^ ^]^ and citric acid‐modified TiO_2_.^[^ ^267^ ^]^ Ultimately, post‐treatment with polymers or functionalization improves dispersion and long‐term stability. For instance, polyaniline‐coated TiO_2_ showed less aggregation, while CuO treated with PVA reduced its particle size from 30 to 15 nm.^[^ ^241^ ^]^

## 13 Conclusion

Various chemical and inorganic modifiers can be used to alter the surface or structure of distinct nanoparticles. Recent research found organic modifiers superior to inorganic modifiers due to their maximum capacity, stability, and nontoxic behavior. In this review, the authors tried to accumulate information on how organic modifiers can bring about structural change in different nanoparticle surfaces. Sometimes, nanoparticles require surface modification so that any further analysis can be done smoothly. In this case, organic modifiers are very helpful in the surface modification of the nanoparticles and provide the necessary structure. This review work will help the researcher to determine the suitable organic modifiers for the particular nanoparticles for their structure modification under optimal conditions and effective parameters that increase the yield of reactions. This study demonstrates how various organic modifiers, including organic acids, polymers, and surfactants, may efficiently modify the size, shape, crystal structure, and dispersion of metal oxides. This review focused on the surface modification of six chosen nanometal oxides in contrast to earlier findings that concentrate on specific modifiers or complex modification methods. This work also highlighted the structural condition of the nanoparticle surface after modification and provided the necessary XRD, SEM, and TEM data for the modified surface. To guarantee smoother modification, this field's present research and development aims to expand the usage of organic modifiers and overcome existing challenges. A deeper comprehension of the process, as well as its operating parameters, may lead to more opportunities for the usage of organic modifiers.

## Conflict of Interest

The authors declare no conflict of interest.

## Author Contributions

**Taskiya Akter** and **Asiful Islam** collected the data and wrote the draft and original manuscript. **Md. Sahadat Hossain** conceived and designed the review, analyzed the data, and assisted in writing the manuscript. **Md. Kawcher Alam** and **Abdullah Al Miad** assisted in collecting data. **Samina Ahmed** supervised the overall work and managed the required facilities.
